# Performance of an Electronic Health Record–Based Automated Pulmonary Embolism Severity Index Score Calculator: Cohort Study in the Emergency Department

**DOI:** 10.2196/58800

**Published:** 2025-01-20

**Authors:** Elizabeth Joyce, James McMullen, Xiaowen Kong, Connor O'Hare, Valerie Gavrila, Anthony Cuttitta, Geoffrey D Barnes, Colin F Greineder

**Affiliations:** 1 Department of Internal Medicine, University of Michigan, Ann Arbor, MI, United States; 2 Department of Internal Medicine, University of San Francisco, San Francisco, CA, United States; 3 Department of Cardiology, University of Michigan, Ann Arbor, MI, United States; 4 Department of Emergency Medicine, University of Michigan, Ann Arbor, MI, United States; 5 Michigan Program on Value Enhancement (MPrOVE), University of Michigan, Ann Arbor, MI, United States

**Keywords:** pulmonary embolism, low-risk pulmonary embolism, risk, artery, pulmonary embolism severity index, clinical decision support, emergency department, hospital, lung, blood, clot, clotting, cardiovascular, index, score, measure, scale, tomography, image, imaging, PESI, CDS, ED

## Abstract

**Background:**

Studies suggest that less than 4% of patients with pulmonary embolisms (PEs) are managed in the outpatient setting. Strong evidence and multiple guidelines support the use of the Pulmonary Embolism Severity Index (PESI) for the identification of acute PE patients appropriate for outpatient management. However, calculating the PESI score can be inconvenient in a busy emergency department (ED). To facilitate integration into ED workflow, we created a 2023 Epic-compatible clinical decision support tool that automatically calculates the PESI score in real-time with patients’ electronic health data (ePESI [Electronic Pulmonary Embolism Severity Index]).

**Objective:**

The primary objectives of this study were to determine the overall accuracy of ePESI and its ability to correctly distinguish high- and low-risk PESI scores within the Epic 2023 software. The secondary objective was to identify variables that impact ePESI accuracy.

**Methods:**

We collected ePESI scores on 500 consecutive patients at least 18 years old who underwent a computerized tomography-pulmonary embolism scan in the ED of our tertiary, academic health center between January 3 and February 15, 2023. We compared ePESI results to a PESI score calculated by 2 independent, medically-trained abstractors blinded to the ePESI and each other’s results. ePESI accuracy was calculated with binomial test. The odds ratio (OR) was calculated using logistic regression.

**Results:**

Of the 500 patients, a total of 203 (40.6%) and 297 (59.4%) patients had low- and high-risk PESI scores, respectively. The ePESI exactly matched the calculated PESI in 394 out of 500 cases, with an accuracy of 78.8% (95% CI 74.9%‐82.3%), and correctly identified low- versus high-risk in 477 out of 500 (95.4%) cases. The accuracy of the ePESI was higher for low-risk scores (OR 2.96, *P*<.001) and lower when patients were without prior encounters in the health system (OR 0.42, *P*=.008).

**Conclusions:**

In this single-center study, the ePESI was highly accurate in discriminating between low- and high-risk scores. The clinical decision support should facilitate real-time identification of patients who may be candidates for outpatient PE management.

## Introduction

While many patients with acute pulmonary embolism (PE) are at increased risk of cardiovascular complications, a significant minority have a low risk of short-term adverse outcomes and no requirement for hospital-level therapies [1]. There is now strong evidence to recommend outpatient management of PE patients identified as low-risk using tools like the Pulmonary Embolism Severity Index (PESI) [2-5].

The PESI score combines demographics (age and sex), comorbidities (history of cancer, heart failure, and chronic lung disease), vital signs (temperature, heart rate, respiratory rate, blood pressure, oxygen saturation), and mental status to stratify acute PE patients according to 30 days all-cause mortality, with classes I (PESI score <66) and II (PESI score 66‐86) being categorized as “low-risk” [2,6]. Despite multiple society-backed guidelines recommending that these patients with low-risk acute PE be provided anticoagulation and discharged directly from the emergency department (ED) [7-9], a recent analysis of over 60,000 cases of acute PE in the US found that less than 4% of patients were managed in the outpatient setting [10]. While there seems to be some variation at the hospital system level, the rate of outpatient management in the US remains substantially lower than in Canada and parts of Europe [11-13].

One potential barrier to increasing outpatient management of low-risk PE is identifying appropriate patients [5,14,15]. Although there are risk-stratification tools, these can be cumbersome to use in a real-world setting. The PESI score, while recommended by guidelines, incorporates 11 clinical variables that have different weights, making it cumbersome to calculate. This may be exacerbated by physician uncertainty regarding specific elements of the score—for example, what constitutes history of cancer?—and the fact that the score can change over the course of ED evaluation, that is, due to changes in mental status and vital signs. A group from the Kaiser Permanente health system previously reported an automated clinical decision support (CDS) tool, which calculates the PESI score based on data already captured in the electronic health record [16]. While found to be highly accurate in predicting patient risk category, that tool was specific to the Kaiser Permanente Epic-based electronic health record (EHR), which was implemented in 2005 and included health-system-specific features, like an improved problem list.

To promote PESI use in our hospital and facilitate more consistent implementation across other health systems, we created a new CDS within our 2023 Epic-based EHR featuring an electronic PESI calculator, or “ePESI” that automatically calculates a patient’s PESI score when a computerized tomography-pulmonary embolism (CT-PE) is ordered. Systematic reviews of CDS system accuracy and use identify poor data quality, poor integration into workflow, patient complexity, inadequate CDS testing, and inadequate user training as negatively impacting the agreement between CDS recommendations and provider decisions [17-20]. In an attempt to account for these limitations, our ePESI underwent over 1 year of testing and multiple revisions. The aim of this study was to determine how well our ePESI captures specific electronic elements from the EHR demonstrating the overall accuracy of the ePESI and its ability to discriminate between patients who would be high- and low-risk if they had a PE, based on PESI score.

## Methods

### Development of the ePESI

The ePESI score was calculated automatically from data available in the EHR. Comorbidities were taken from the patient’s active problem list and past medical history using preestablished SNOMED (Systematized Nomenclature of Medicine) hierarchical concept codes (Multimedia Appendix 1). Vital sign criteria were assigned using peak vital signs from the patient’s Flowsheet (ie, structured ED nursing documentation)—that is, highest heart rate and respiratory rate, lowest systolic blood pressure, etc. The PESI element “oxygen saturation <90%” was considered positive if the patient had a documented pulse oximetry reading of <90%, or if they had been placed on >2 L/min of oxygen, >2 L/min above their home O2 requirement, or a respiratory-assist device such as BiPAP (bilevel positive airway pressure) or CPAP (continuous positive airway pressure), venturi mask, high-flow catheter, or mechanical ventilator. Altered mental status was defined as Glasgow Coma Scale (GCS) <14 in structured ED nursing documentation or “Altered Mental Status” in the patient’s chief concern at ED presentation.

### Study Design, Setting, and Population

**Figure 1.**
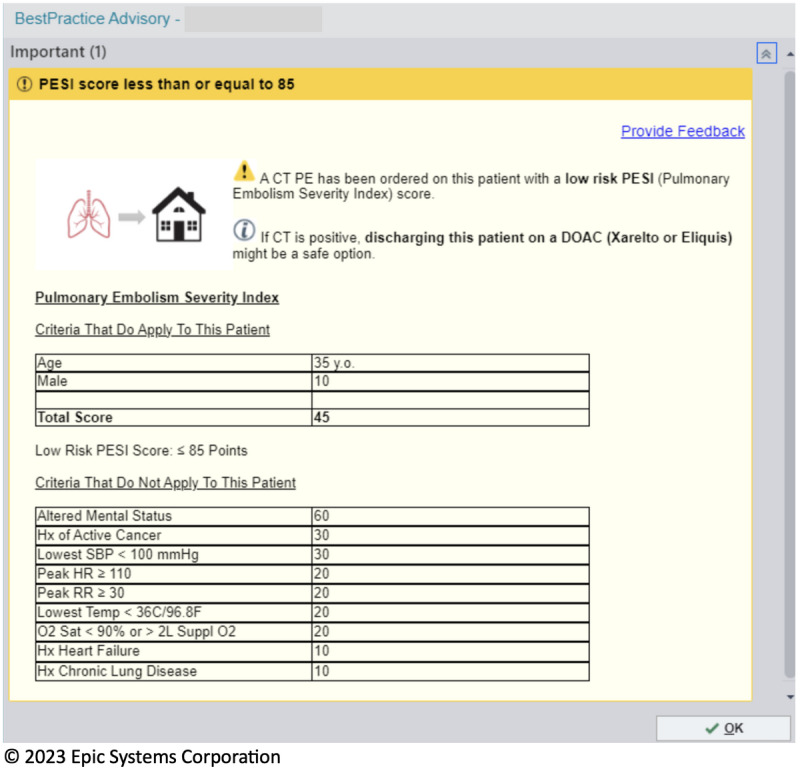
Image of the best practice alert presented to clinicians, depicting the Electronic Pulmonary Embolism Severity Index (ePESI) score. CT: computed tomography; DOAC: direct oral anticoagulant; HR: heart rate; Hx: history; O2 Sat: oxygen saturation, as measured by pulse oximetry; PE: pulmonary embolism; RR: respiratory rate; SBP: systolic blood pressure; Suppl O2: supplemental oxygen; Temp: temperature.

In March 2022, a “pre-commitment” strategy was implemented in our ED to encourage outpatient management of low-risk acute PE [21]. The ePESI was calculated in all patients in whom a CT-PE was ordered. In patients with ePESI <86, the overall score and the individual elements were displayed to the treating clinician at the time of CT-PE order, encouraging them to consider outpatient management if a PE was ultimately diagnosed (Figure 1). In this study, we examined ePESI results in 500 consecutive patients who underwent CT-PE between January 3 and February 15, 2023. We included all patients, regardless of their CT-PE scan result, because the presence or absence of a PE should not influence the CDS accuracy when sampling electronic health data.

### Ethical Considerations

This study was exempt from the institutional review board at the University of Michigan given that it was in regard to procedures rather than individual patients (HUM00217018). Participant consent was waived by the institutional review board. Permission was obtained from Epic to display any images of the ePESI.

### Physician PESI (pPESI)

Two independent, medically-trained reviewers (EJ and JM) abstracted data from the EHR on all patients and calculated a physician PESI (pPESI) score using a standardized abstraction form (Multimedia Appendix 2). Both data abstraction and pPESI calculation occurred retrospectively, after all 500 cases had been identified. While reviewers could not be blinded to the study hypothesis, they were blinded to the ePESI score and to each other’s pPESI scores. In cases where the 2 reviewers did not agree, a third reviewer (CO) abstracted data and calculated another blinded pPESI.

### Statistical Analysis

Descriptive statistics, reported as proportions, were used to describe relevant characteristics of the patient population and their PESI scores. All cases in which the ePESI and pPESI did not agree underwent retrospective chart review to identify the discrepancy. Statistical analysis was used to compare ePESI and pPESI, with pPESI as the gold standard. The ePESI accuracy was computed via binomial test and reported with the 95% CI. Dichotomous predictors of ePESI accuracy were identified with a priori hypotheses and evaluated using odds ratios (OR). The OR was computed using logistic regression with continuous scoring. The error rates of individual ePESI components were reported as proportions and evaluated with ANOVA. *P* values of <.05 were considered statistically significant. Inter-rater reliability was reported as a Cohen κ coefficient. All data analysis was conducted with SAS (version 9.4; SAS Institute).

## Results

Of the 500 consecutive patients in whom ePESI results were evaluated, 203 (40.6%) patients had low-risk scores (ePESI<86) and 297 (59.4%) patients with high-risk scores (ePESI≥86) (Table 1). The most frequent risk class was class V (133/500, 26.6%), followed by class I (112/500, 22.4%). A total of 90% of patients (450/500) had had at least 1 encounter in the health institution’s EHR prior to their index ED visit, whereas the remaining 10% of patients presented to the ED for their first entry into the health institution’s EHR. For those with prior encounters, the median time between index ED visit and previous EHR entry was 14 days (IQR 4‐50.7). Of the 500 study patients undergoing CT-PE, a pulmonary embolism was diagnosed in 47 patients (9.4%), of which 40 patients (85.1%) had an acute PE and 7 patients (14.9%) had subacute PE, chronic PE, or an equivocal study. Interrater reliability for the pPESI score was high, with Cohen κ coefficient of 0.98 (95% CI 0.97‐0.98).

**Table 1.** Patient characteristics.

Characteristics	Values
Age (years), mean (SD)	58.2 (17.7)
Biologic male, n (%)	219 (43.8)
Comorbidities, n (%)	
History of cancer	185 (37)
History of heart failure	88 (17.6)
History of chronic lung disease	205 (41)
High risk versus low risk, n (%)	
High risk	297 (59.4)
Low risk	203 (40.6)
Class of risk, n (%)	
Class I	112 (22.4)
Class II	91 (18.2)
Class III	84 (16.8)
Class IV	80 (16)
Class V	133 (26.6)
First visit at health institution, n (%)	
Yes	50 (10)
No	450 (90)
CT-PE^a^ scan report result, n (%)	
Acute PE^b^	40 (8)
Chronic PE	6 (1.2)
Acute on chronic PE	1 (0.2)
Other	3 (0.6)
No PE (negative)	451 (90.2)

a CT-PE: computed tomography scan-pulmonary embolism.

b PE: pulmonary embolism.

**Figure 2.**
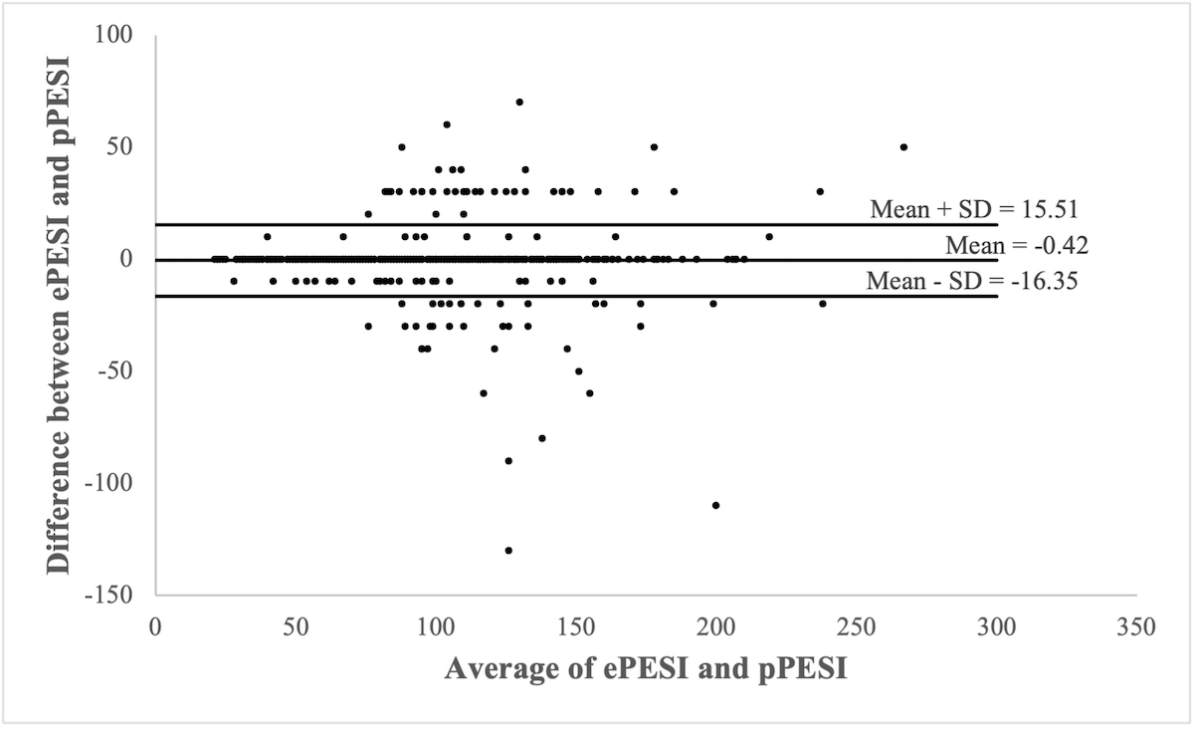
Bland-Altman plot demonstrating the agreement between the absolute value of ePESI and pPESI. ePESI: Electronic Pulmonary Embolism Severity Index; pPESI: Physician Pulmonary Embolism Severity Index.

The ePESI and pPESI scores matched exactly in 394 out of 500 cases, for an accuracy of 78.8% (95% CI 74.9%‐82.3%) (Figure 2). For the subset of patients with an acute PE found on CT-PE, the accuracy was 82.5% (95% CI 67.2%‐92.7%) (Tables2^4^). The ePESI correctly categorized risk class (I-V) in 442 out of 500 (88.4%) cases and correctly identified high- or low-risk in 477 out of 500 (95.4%) cases. For cases where the ePESI and pPESI did not agree on high- or low-risk, the ePESI incorrectly identified cases as low-risk in 15 out of 23 (65.2%) and incorrectly identified cases as high-risk in 8 out of 23 (34.8%) patients. The odds of the ePESI and pPESI agreeing increased if scores were low-risk by pPESI (OR 2.96, CI 1.78-4.91, *P*<.001) (Table 5). The odds of the ePESI and pPESI agreeing decreased if the index ED visit represented the patient’s first entry in the institution’s EHR (OR 0.42, CI 0.214-0.788, *P*=.008).

**Table 2.** Comparison of Electronic Pulmonary Embolism Severity Index (ePESI) and Physician Pulmonary Embolism Severity Index (pPESI).

	pPESI correct	pPESI incorrect
ePESI correct	394	9
ePESI incorrect	92	5

**Table 3.** Electronic Pulmonary Embolism Severity Index (ePESI) accuracy for full cohort and positive computerized tomography-pulmonary embolism (CT-PE) cohort.

	Number, n (%)	Accuracy, % (95% CI)
Full cohort	500 (100)	78.8 (74.9‐82.3)
Positive CT-PE	40 (8)	82.5 (67.2‐92.7)

**Table 4.** Electronic Pulmonary Embolism Severity Index (ePESI) performance for cohorts.

Cohort	Correct, n (%)	Incorrect, n (%)
High- versus low-risk PESI^a^	477 (95.4)	23 (4.6)
Class of risk	442 (88.4)	52 (11.6)

a PESI: Pulmonary Embolism Severity Index.

**Table 5.** Logistic regression modeled Physician Pulmonary Embolism Severity Index (pPESI) and Electronic Pulmonary Embolism Severity Index (ePESI) result agreeing by dichotomous predictor.

Variable	Odds ratio	95% CI	*P* value
Highlow (low vs high)	2.96	1.78‐4.91	<.001
First time at health system (yes vs no)	0.41	0.21‐0.79	.008

When considering individual components of the ePESI, age was most often correctly identified (n=500, 100% correct), while the history of cancer was least often correctly identified (n=459, 91.8% correct) (Table 6). Sex was incorrectly identified by ePESI in 1 (0.2%) case, for a transgender patient in which gender and sex assigned at birth had been conflated in the EHR.

**Table 6.** Comparison of errors. Number of patients with each type of error is indicated above each bar. Patients could have more than 1 type of error.

Types^a^	Variable, n (%)
Cancer error	42 (8.4)
HF^b^ error	16 (3.2)
CLD^c^ error	27 (5.4)
Vitals error	23 (4.6)
Other	1 (0.2)

a *P* value<.001.

b HF: heart failure.

c CLD: chronic lung disease.

## Discussion

### Overview

In this single-center cohort study, we demonstrated the accuracy of an EHR-based ePESI calculator built into the Epic 2023 system. The ePESI was particularly effective at discriminating between low- and high-risk cases, with 95% (477/500) correctly scoring as high- or low-risk as compared with a gold standard of multiple physician review. The accuracy of our ePESI calculator is similar to that of the PESI CDS tool developed by the Kaiser Permanente health system in 2015 [16], but it has the advantage of being compatible with Epic 2023 software and minimally reliant on health system-specific features. This should enable straightforward transfer to other health systems using Epic-based EHRs. Since 2015, Epic has surpassed Cerner as the most common EHR in the United States, and comprises 36% of the market share as of 2023 [22,23]. Epic is also gaining international use [24]. Given the increasingly widespread use of Epic EHR and interest in implementing outpatient management pathways for low-risk PE patients, we anticipate that the ePESI calculator built into the Epic 2023 software that can be readily exported to other health systems will be of interest to many EDs around the world.

Apart from the overall performance of the ePESI calculator, our study has several interesting findings with respect to automated risk score calculation. First, the ePESI calculator performed better when the ePESI score was low risk and in patients who had been seen within our health system previously. These findings are not entirely unexpected, as most ePESI errors were due to incorrect scoring of patient comorbidities (ie, cancer, heart failure, and lung disease), which were more common in high-risk cases and more likely to be inaccurate for patients whose index ED visit was their first presentation to our health system. Indeed, many errors stemmed from elements of the medical history not being entered or fully updated at the time of CT-PE order and ePESI calculation. While less problematic than comorbidities, vital signs were occasionally measured prior to CT-PE order but entered into the EHR after the ePESI calculator had been triggered to automatically calculate the ePESI score, causing some errors. These issues may be less problematic if health systems set the ePESI calculator to calculate the score after a PE diagnosis is made, rather than at the time of CT-PE order. Nonetheless, real-time updating of problem lists and vital signs is outside the capability of most EDs, and it is likely that some errors are simply unavoidable. The incorrect scoring of sex for 1 transgender patient in our cohort exemplifies the near impossibility of accurately evaluating the PESI score for every possible ED patient.

To some extent, these observations also highlight the limitations of the PESI score itself, and more generally, any risk stratification metric developed without consideration for contemporary health care technology. Several terms incorporated into both the PESI and simplified PESI scores—for example, history of cancer and heart failure—are vague and fail to account for the complexity of current medical practice or the ability to accurately capture it in the EHR. For example, how should we score patients with non-metastatic skin cancer or heart failure with recovered ejection fraction? What about patients who are placed immediately on supplemental oxygen and never have a charted SpO_2_ less than 90%? Best practice alerts and electronic CDS tools are increasingly used in medical practice, and as technology continues to evolve, it is likely that future risk stratification metrics will need to be developed with an eye toward what can be accurately quantified by the EHR, communicated to clinicians in real time, and evaluated retrospectively for quality improvement or research purposes. In this sense, the development of this ePESI calculator and the demonstration of its utility across institutions may pave the way for more standardized methods of disease stratification and corresponding management recommendations.

### Principal Results

This study demonstrates the accuracy of our ePESI calculator, which has 95% accuracy in discriminating between high- and low-risk PESI scores. The odds of the ePESI and pPESI agreeing increased (OR 2.96, *P*<.001) if a patient had a low-risk PESI score and decreased if it was the patient’s first time in the health system (OR 0.41, *P*=.008). Most errors were due to incongruities with the problem list, of which cancer is the most common error (91.8% correct).

### Limitations

Our study had several strengths, including the use of blinded assessment of the ePESI score and the inclusion of consecutive patients undergoing CT-PE. In addition, there are several limitations of this study that must be considered. The first is that the SNOMED hierarchical concept codes used by the ePESI are somewhat specific to the health system where the study was conducted. The concept codes are adapted from general Epic concept codes, so we anticipate the transfer of our concept codes to other institutions will be straightforward. However, some degree of adjustment may be necessary and performance may differ at other sites. Indeed, efforts to adapt and validate the ePESI in other locoregional hospitals are ongoing. A second limitation is that the pPESI was calculated retrospectively, rather than in real time by providers ordering the CT-PE. We adjusted for this with 2 independent reviewers, blinded to the ePESI score and each other’s pPESI score, to mitigate potential bias from the retrospective calculation. Additionally, reviewers used a standardized data abstraction approach designed to mimic the information providers have access to and likely use in the ED. A third limitation is that this study was conducted in a cohort of patients with a variety of conditions, not just those with PE. The purpose of this study was to evaluate the ePESI accuracy when it automatically sampled comorbidities, demographics, and vital signs, none of which are dependent on the patient’s diagnosis. There is no reason to conclude that EHR accuracy should meaningfully differ for patients with and without a PE. The purpose of this study was not to influence clinical practice or predict patient outcomes, although this should be evaluated in a future study now that we have demonstrated the accuracy of the ePESI in sampling electronic health data. Additionally, we conducted a sensitivity analysis in the subset of patients with acute PE and found the ePESI accuracy to be comparable to our overall cohort. The number of patients with acute PE in our study was small, but it is unlikely, although not impossible, that the accuracy of the ePESI will be dramatically altered in a larger cohort of patients with acute PE. A final limitation is that we were unable to account for all variables that influence ePESI and pPESI agreement. As discussed in the introduction paragraph, systemic reviews of generalized CDS systems suggest that patient complexity, data quality, poor integration, inadequate testing, and inadequate training are all associated with poor CDS-provider agreement [17-20]. We attempted to minimize these by using a standardized protocol for the pPESI reviewers, as described in the methods paragraph, and updated or ePESI based on provider feedback before we formally tested it. However, it is possible these still affected our ePESI and pPESI agreement.

### Comparison With Prior Work

Our study demonstrates a PESI score calculator with similar accuracy to a calculator described by Vinson et al [16] in the Kaiser Permanente system in 2015. However, our study has the advantage of describing a calculator that can be readily and immediately exported to other health centers. Our ePESI was developed within the Epic 2023 software, as compared with the 2015 software, and is minimally reliant on features specific to our health system. The Kaiser Permanente ePESI depends on the improved problem list, which limits direct translation to other health systems. Additionally, our ePESI was programmed to calculate and alert providers in the ED for easy and direct integration into clinical practice. To our knowledge, these are the only 2 studies evaluating a CDS tool that automatically calculates PESI scores using patient data from the EHR.

### Conclusions

In conclusion, we created a CDS that is highly reliable at discriminating between low- and high-risk PE patients based on the PESI score. It is most accurate in the low-risk PESI score population, which is its intended target for clinical use. Our CDS has the potential to be exported to other health systems and implemented more widely to increase the discharge of low-risk PE patients who do not otherwise require hospitalization.

## Supplementary material

10.2196/58800 Multimedia Appendix 1 Table S1 SNOMED hierarchical concepts for comorbidities.

10.2196/58800 Multimedia Appendix 2 Table S2 Forecasted age-standardized mortality rates for asthma-related deaths by 2035.

## ABBREVIATIONS

BiPAP: bilevel positive airway pressure
CDS: clinician decision support
CPAP: continuous positive airway pressure
CT-PE: computerized tomography-pulmonary embolism
ED: emergency department
EHR: electronic health record
ePESI: Electronic Pulmonary Embolism Severity Index
GCS: Glasgow Coma Scale
OR: odds ratio
PE: pulmonary embolism
PESI: Pulmonary Embolism Severity Index
pPESI: Physician Pulmonary Embolism Severity Index
SNOMED: Systematized Nomenclature of Medicine
