# Supplementary material for: Performance of an Electronic Health Record–Based Automated Pulmonary Embolism Severity Index Score Calculator: Cohort Study in the Emergency Department
Source: JMIR Med Inform. 2025 Jan 20;13:e58800. doi: 10.2196/58800 (PMC11769779; doi:10.2196/58800)
Supplement: Multimedia Appendix 1 [file medinform-v13-e58800-s001.docx]

Multimedia Appendix Table S1: SNOMED hierarchical concepts for comorbidities.

| Comorbidity | Subset |
| --- | --- |
| Cancer | - Snomed Hierarchical Concept of “MALIGNANT NEOPLASTIC DISEASE”( SNOMED#363346000) OR - Snomed Hierarchical Concept of “MERKEL CELL CARCINOMA”( SNOMED#253001006) OR - Snomed Hierarchical Concept of “NEUROENDOCRINE TUMOR”( SNOMED#255046005 ) OR - Snomed Hierarchical Concept of “ADENOCARCINOMA OF LUNG”( SNOMED#254626006 ) OR - Snomed Hierarchical Concept of “ADENOCARCINOMA OF LUNG, STAGE IV”( SNOMED#424993006 ) OR - Snomed Hierarchical Concept of “PRIMARY ADENOCARCINOMA OF LUNG”( SNOMED#707451005 ) AND - NOT Snomed Hierarchical Concept of “MALIGNANT NEOPLASM OF SKIN”( SNOMED#372130007) |
| Chronic Lung Disease (CLD) | - Snomed Hierarchical Concept of “CHRONIC LUNG DISEASE”( SNOMED#413839001) OR - Snomed Hierarchical Concept of “HISTORY OF CHRONIC LUNG DISEASE”( SNOMED#414415007 ) OR - Snomed Hierarchical Concept of “DEPENDENCE ON HOME VENTILATOR”( SNOMED#60631000119109 ) OR - Snomed Hierarchical Concept of “REQUIRES CONTINUOUS HOME OXYGEN SUPPLY”( SNOMED#716366009) OR - Snomed Hierarchical Concept of “RAPID-ONSET CHILDHOOD OBESITY, HYPOTHALAMIC DYSFUNCTION, HYPOVENTILATION, AUTONOMIC DYSREGULATION SYNDROME”( SNOMED#773663004) OR - Snomed Hierarchical Concept of “ASTHMA”( SNOMED#195967001 ) OR - Snomed Hierarchical Concept of “CHRONIC RESPIRATORY FAILURE”( SNOMED#39871006 ) OR - Snomed Hierarchical Concept of “PULMONARY SARCOIDOSIS”( SNOMED#24369008 ) OR - Snomed Hierarchical Concept of “INTERSTITIAL LUNG DISEASE”( SNOMED#233703007 ) OR - Snomed Hierarchical Concept of “HYPERSENSITIVITY PNEUMONITIS NOS”( SNOMED#195994002) OR - Snomed Hierarchical Concept of “CYSTIC FIBROSIS”( SNOMED#190905008 ) OR - Snomed Hierarchical Concept of “SARCOIDOSIS”( SNOMED#31541009 ) OR - Snomed Hierarchical Concept of “SIMPLE CHRONIC BRONCHITIS”( SNOMED#61937009 ) |
| Heart Failure (HF) | - Snomed Hierarchical Concept of “HEART FAILURE”( SNOMED#84114007) OR - Snomed Hierarchical Concept of “H/O: HEART FAILURE”( SNOMED#161505003) OR - Snomed Hierarchical Concept of “HEART FAILURE WITH NORMAL EJECTION FRACTION”( SNOMED#446221000) OR - Snomed Hierarchical Concept of “HEART FAILURE WITH REDUCED EJECTION FRACTION”( SNOMED#703272007) OR - Snomed Hierarchical Concept of “CARDIOMYOPATHY”( SNOMED#85898001) OR - Ischemic cardiomyopathy [288097] OR - Cardiomyopathy, ischemic [288098] |
