# Supplementary material for: Performance of an Electronic Health Record–Based Automated Pulmonary Embolism Severity Index Score Calculator: Cohort Study in the Emergency Department
Source: JMIR Med Inform. 2025 Jan 20;13:e58800. doi: 10.2196/58800 (PMC11769779; doi:10.2196/58800)
Supplement: Multimedia Appendix 2 [file medinform-v13-e58800-s002.docx]

Multimedia Appendix Table S2. Protocol for abstracting data for the pPESI. ED: emergency department; PESI: pulmonary embolism severity index; HR: heart rate; CT-PE: computerized tomography-pulmonary embolism; RR: respiratory rate; SBP: systolic blood pressure; GCS: Glasgow coma scale; O2 sat: oxygen saturation as measured by a pulse oximeter; L: liters.

| **PESI Field:** | **Criteria:** |
| --- | --- |
| Age | Please check to make sure birthday is not between date of data abstraction and the date of ED encounter |
| Gender (Male=+10) | Biologic sex |
| History of active Cancer (+30) | In ED provider note: check active problem list IN THE ED NOTE, past medical history (usually have to expand), and first line.  If the problem list or past medical history list is missing, look at the problem list/past medical history of the note just prior to the ED visit.  Criteria included   - Malignant neoplasm, malignant [organ/site] - Cancer - Carcinoma - Sarcoma - Leukemia - Lymphoma - ALL, AML, CML, CLL   Criteria excluded   - Benign - Family history of a cancer but no personal history of a cancer - In remission - Non-metastatic skin cancer - Non-metastatic cervical cancer |
| History of Heart Failure (+10) | In ED provider note: check active problem list IN THE ED NOTE, past medical history (usually have to expand), and first line.  In ED provider note: check active problem list IN THE ED NOTE, past medical history (usually have to expand), and first line. You do not need to dig back through any other notes!  If the problem list or past medical history list is missing, look at the problem list/past medical history of the note just prior to the ED visit.  Criteria included   - Hypertensive heart failure (including renal/malignant) - Congestive heart failure - Chronic heart failure (can include acute exacerbation) - Cardiomyopathy (dilated, ischemic, etc.)   Criteria excluded   - Acute heart failure - Right heart strain - Pulmonary embolism with acute cor pulmonale |
| History of Chronic Lung Disease (+10) | In ED provider note: check active problem list IN THE ED NOTE, past medical history (usually have to expand), and first line.  If the problem list or past medical history list is missing, look at the problem list/past medical history of the note just prior to the ED visit.  Criteria included (not limited to these conditions, but these are new additions since PESI calculator was revised)   - Adult pulmonary Langerhans cell - Allergic bronchopulmonary aspergillosis (ABPA) - Asbestosis - Asthma - Bronchiectasis - Chronic airway obstruction - Chronic Bronchitis (including chronic bronchitis with acute exacerbation and many other iterations) - Chronic Obstructive Pulmonary Disease (COPD) - Chronic respiratory conditions due to fumes and vapors - Chronic Respiratory Failure (anything with this in it) - Cryptogenic organizing pneumonia - Emphysema (including emphysematous bleb) - Extrinsic allergic alveolitis - Hypersensitivity pneumonitis - Idiopathic interstitial pneumonia? (anything with these three words +/- others) - Idiopathic nonspecific interstitial pneumonitis (NSIP) - Idiopathic pulmonary fibrosis - Idiopathic pulmonary hemosiderosis - Interstitial Lung Disease (anything with this) - Lymphangioleiomyomatosis - Pneumoconiosis (any iteration) - Pneumonopathy - Pulmonary fibrosis - Pulmonary proteinosis - Pulmonary Sarcoidosis - Systemic sclerosis lung disease   Exclude:   - Acute bronchitis - Acute respiratory conditions due to fumes and vapors - Acute respiratory failure - Chronic thromboembolic pulmonary hypertension (CTEPH) - Obstructive Sleep Apnea (OSA) - Empyema - Lung cancer (any. This should go under cancers, not lung disease) - Pleurisy - Pneumonia - Pneumonitis due to solids and liquids - Pneumothorax - Pulmonary congestion - Pulmonary hypertension (pHTN) |
| Peak HR>=110 (+20) | See flowsheet*, must be before CT-PE ORDER but NOT from a clinic visit right before ED visit |
| Peak RR>=30 (+20) | See flowsheet*, must be before CT-PE ORDER but NOT from a clinic visit right before ED visit |
| Lowest Temperature <36C/96.8F (+20) | See flowsheet*, must be before CT-PE ORDER but NOT from a clinic visit right before ED visit |
| Lowest SBP <100mmHg (+30) | See flowsheet*, must be before CT-PE ORDER but NOT from a clinic visit right before ED visit |
| Altered Mental Status (+60) | - GCS UNDER 14 (under “Flowsheets*”) before CT-PE ORDER - AMS or Altered Mental Status in Chief Concern |
| O2 sat <90% or >2L suppl O2 (+20) | See flowsheet*, must be before CT-PE ORDER but NOT from a clinic visit right before ED visit  Remember, being on 2L does NOT count. It needs to be >2L.  Additional inclusion criteria:   - Check ED note for documentation of patient being on >2L at baseline/on arrival - Patient on higher-level oxygenation device (ventilation, BiPAP, non-rebreather, Venturi mask, etc.)  before CT-PE order |
